# The PDZ Domain as a Complex Adaptive System

**DOI:** 10.1371/journal.pone.0000953

**Published:** 2007-09-26

**Authors:** Alexei Kurakin, Andrzej Swistowski, Susan C. Wu, Dale E. Bredesen

**Affiliations:** The Buck Institute for Age Research, Novato, California, United States of America; University of Washington, United States of America

## Abstract

Specific protein associations define the wiring of protein interaction networks and thus control the organization and functioning of the cell as a whole. Peptide recognition by PDZ and other protein interaction domains represents one of the best-studied classes of specific protein associations. However, a mechanistic understanding of the relationship between selectivity and promiscuity commonly observed in the interactions mediated by peptide recognition modules as well as its functional meaning remain elusive. To address these questions in a comprehensive manner, two large populations of artificial and natural peptide ligands of six archetypal PDZ domains from the synaptic proteins PSD95 and SAP97 were generated by target-assisted iterative screening (TAIS) of combinatorial peptide libraries and by synthesis of proteomic fragments, correspondingly. A comparative statistical analysis of affinity-ranked artificial and natural ligands yielded a comprehensive picture of known and novel PDZ ligand specificity determinants, revealing a hitherto unappreciated combination of specificity and adaptive plasticity inherent to PDZ domain recognition. We propose a reconceptualization of the PDZ domain in terms of a complex adaptive system representing a flexible compromise between the rigid order of exquisite specificity and the chaos of unselective promiscuity, which has evolved to mediate two mutually contradictory properties required of such higher order sub-cellular organizations as synapses, cell junctions, and others – organizational structure and organizational plasticity/adaptability. The generalization of this reconceptualization in regard to other protein interaction modules and specific protein associations is consistent with the image of the cell as a complex adaptive macromolecular system as opposed to clockwork.

## Introduction

Protein interaction modules, such as PDZ, SH3, WW, EH, SH2 and other domains, mediate protein-protein interactions by recognizing and binding short and usually linear peptide epitopes within their interacting partners [1]–[4]. The importance of this particular class of protein interactions *in vivo* is underscored by the estimates suggesting that a substantial fraction of all specific protein interactions in the cell may involve peptide recognition domains [3], [5].

PDZ domain is a prototypical and one of the best-characterized protein interaction modules. Approximately 90 amino acids long, PDZ domain was first discovered as sequence repeats in the primary structures of the post-synaptic density 95 (PSD95), disk-large (Dlg) and zona occludens-1 (ZO-1) proteins [6]. Later it was identified in many other proteins and the first draft of the human genome ranked the PDZ domain family as number 19 among the most abundant domain families [7]. More than 400 different PDZ domains are currently estimated to exist in humans or in mice. PDZ domains often occur in multiple copies within proteins, as well as in various combinations with other types of protein interaction modules and/or functional domains. The abundance of PDZ domains in metazoan genomes together with the scarcity of canonical PDZ domains in non-metazoans indicates a possibly critical function of PDZ domains in multicellular organization [8].

While able to interact with internal amino acid sequences properly constrained within secondary structure, in their canonical and by far the most common mode of interaction PDZ domains recognize and bind short specific sequences at the extreme C-termini of their interacting partners [9]. Recognition of C-termini represents a form of non-invasive interaction well suited to mediate organization of transport, localization, sorting and spatial arrangement of proteins using their individual C-terminal tails recognized and handled by various PDZ domains. Perhaps not surprisingly, many PDZ domain proteins, especially those containing multiple copies of PDZ domains, function as scaffolds at the specialized membrane regions in the cell, where they manage organization and maintenance of large macromolecular complexes, such as signal-processing machinery at post-synaptic densities (PSD) [10], [11].

**Figure 1:**
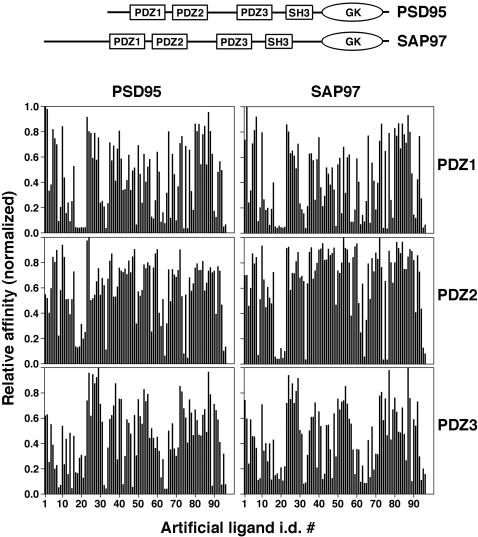
Binding of 95 artificial phage-displayed ligands to six PDZ domains of PSD95 and SAP97. Binding histograms were obtained by individual phage ELISA performed on purified GST fusions of the indicated domains immobilized in micro-titer plate wells [40]. For accurate relative affinity evaluations and cross-domain comparison, the slopes of individual ELISA kinetics were determined and normalized by the highest slope value in each of the six sets shown. The 96^th^ well in each set was loaded with a library aliquot to indicate background. Axis X indicates identification (i.d.) numbers of individual artificial ligands (see individual ligand sequences together with their i.d. numbers in Table S1). Axis Y indicates the normalized relative affinity. The domain organization of the PSD95 and SAP97 proteins is shown above histograms.

Post-synaptic density protein 95 (PSD95) is an archetypal member of the synapse-associated protein (SAP) family of scaffolding molecules comprising PSD95/SAP90, SAP97, SAP102 and PSD93/chapsyn110. SAP proteins function as key organizers that control synaptic composition, organization and function [11], [12]. The members of the SAP family share the same overall domain organization with three N-terminal PDZ domains followed by an SH3 domain and a guanylate homology domain at the C-terminus (Fig. 1). All five domains appear to function as protein interaction modules mediating associations of SAP scaffolds with their multiple interacting partners [11].

Over the 15 years since the discovery of PDZ domains, the biochemistry and structural basis of PDZ domain recognition as well as the biology of PDZ domain-containing proteins have been subjects of numerous studies, which are summarized in a number of reviews [8], [9], [11]. There are, however, three major uncertainties that appear to persist in the PDZ domain field, despite extensive research efforts to clarify them. These are 1) the degree of selectivity of individual PDZ domains, 2) the significance of the ligand residues situated upstream of the last four C-terminal amino acids and 3) the physiological affinity range of PDZ domain interactions.

The first uncertainty is illustrated by the continual but so far failed attempts to classify PDZ domains in accord with their specificities (see the examples of at least five different classifications in Refs. [8], [9], [13]-[15]). It is generally agreed that there are two major classes of PDZ domains – class I PDZ domains recognize and bind the C-termini of proteins conforming to the consensus sequence X-(S/T)-X-(V/I/L)-COOH, while class II PDZ domains interact with the C-terminal consensus X-Φ-X-Φ-COOH, where X is any amino acid and Φ stands for hydrophobic residue [16]. In the last ten years following the identification of these two major classes, PDZ domain classification became increasingly complicated, mainly due to the discoveries that a) there are a number of, and potentially many, distinct PDZ domain specificity classes, b) at least some of the PDZ domains can be classified into more than one class, as they are able to interact with the peptide ligands that do not share a common consensus at their C-termini and c) some of the same class PDZ domains can clearly differentiate between the ligands sharing a class-defining consensus, such as X-(S/T)-X-(V/I/L)-COOH, for example [17], [18]. At the same time, PDZ domains became notorious for their apparent promiscuity [18], [19].

The second major uncertainty pertains to the contribution of the ligand residues that are situated upstream of the last three to four C-terminal amino acids. Since the initial structural studies implicating only the few carboxy-terminal ligand residues in direct interactions with PDZ domains, it has been assumed that the influence of the upstream residues in PDZ ligands is inconsequential for PDZ domain interactions, and the occasional experimental evidence to the contrary is normally regarded as exceptional, relevant only for a particular domain or even a particular domain-ligand pair [15], [20], [21]. The generality of this assumption, however, becomes increasingly questionable as the examples demonstrating involvement of the upstream ligand residues continue to accumulate [17], [22], [23].

The widely diverse affinities reported for PDZ domain-peptide interactions, spanning more than three orders of magnitude, represent another source of confusion. Because in-solution methods, such as fluorescence polarization (FP), tend to estimate PDZ domain-peptide interaction affinities in the low micromolar range, well within the affinity range expected from protein interaction domains mediating transient specific associations inside the cell, they are generally perceived as more trustworthy than the *K_D_* values in the low to medium nanomolar range obtained by solid phase methods, such as surface plasmon resonance (SPR) or enzyme-linked immunosorbent assays (ELISA) [8], [9]. However, since both PDZ domain-containing proteins and pertinent PDZ domain ligands are often found clustered at the specialized membrane regions, such as postsynaptic densities or cellular junctions, a question has been raised as to which method approximates the *in vivo* situation better and what affinities should be considered as physiologically relevant for PDZ domain-mediated interactions [9].

It should be emphasized that the ambiguities detailed above for the PDZ domain family are common, to a larger or smaller degree, to all the peptide recognition domain families [3], [24], thus creating an apparent paradox – how the cell achieves its highly organized state while relying on the molecular interactions of limited selectivity?

To address the above-mentioned questions in a comprehensive manner, we applied a number of novel biochemical and statistical approaches to generate and analyze large populations of peptide ligands for a number of well-studied PDZ domains. The results of this study reveal a hitherto unappreciated combination of specificity and adaptive plasticity inherent to PDZ domain recognition. The complexity of PDZ domain recognition and the seemingly contradictory and/or confusing observations accumulated in the field are reconciled within a novel, if unexpected, image of the PDZ domain emerging as a complex adaptive system evolved to ensure both structure and organizational plasticity of higher order dynamic macromolecular systems such as synapses, cell junctions, and others.

## Results

### Analysis of PDZ domain recognition: artificial ligands

This study capitalizes on distinct advantages of the novel screening format for phage-displayed peptide libraries, target-assisted iterative screening (TAIS), introduced recently and described elsewhere [25], [26]. Omitting competition between individual binders and switching molecular context in target presentation, TAIS allows for selection of specific peptide binders to a given protein target in a wide range of affinities with no false positives, and thus provides unique and unexploited opportunity to generate large datasets for analysis of individual binders on the one-by-one basis, rather than, as is done traditionally, considering population averages (synthetic peptide library screens) [16] or a few of the best binders only (conventional panning) [27], [28]. TAIS was applied to cDNA and random 16-mer peptide libraries in a search for peptide ligands of various PDZ domains of the SAP family of proteins. The relative affinities of the 95 artificial ligands isolated from peptide libraries towards six PDZ domains of the PSD95 and SAP97 proteins are shown in Fig. 1 (see individual ligand sequences together with their i.d. numbers in Table S1).

Visual examination of binding histograms suggests that 1) the recognition specificities of all six domains examined are very similar, albeit not identical; 2) the second domains of both proteins are noticeably more promiscuous than their first and third domains; and 3) the specificities of homologous domains across different proteins appear to be more similar than the specificities of the domains belonging to the same protein. The first two observations are in agreement with the established body of experimental evidence [17], [18], [29], [30]. The third observation suggests a likely evolutionary scenario, in which the SAP family of proteins originated by duplication-divergence of an individual PDZ domain within an ancestor protein, followed by duplication-divergence of a whole protein to generate the family members characterized by overlapping but distinct biological roles/functions [11], [12].

**Figure 2:**
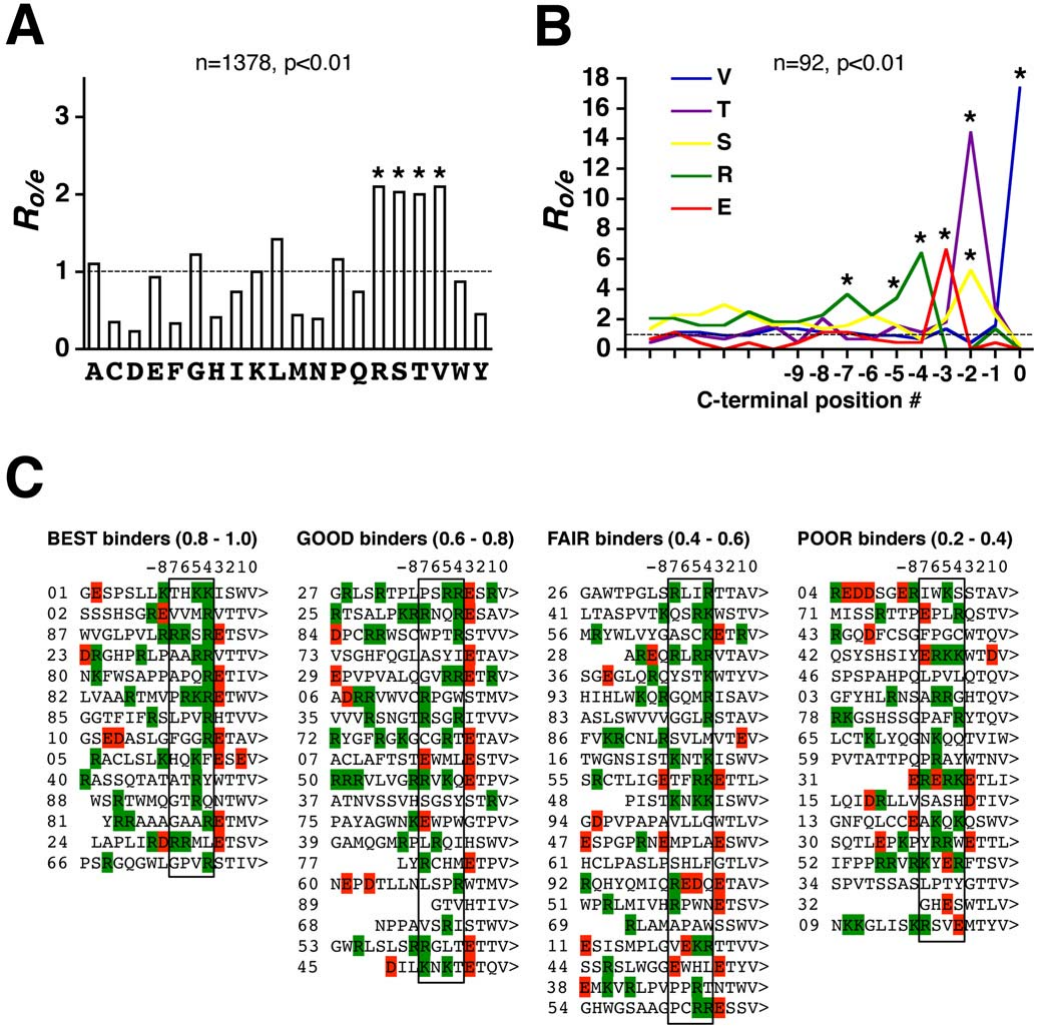
RFP (residue frequency patterning) analysis of artificial ligands. *A*, The observed-to-expected ratios of individual amino acid frequencies within the whole set of artificial peptide ligands isolated from cDNA and random peptide libraries by TAIS using SAP PDZ domains as targets. *B*, The frequency distributions of the indicated amino acids within the last sixteen C-terminal positions of aligned artificial peptide ligands. For both *A* and *B*: axis Y indicates the observed-to-expected frequency ratio values. The dotted line corresponds to the expected frequency value. Statistically significant overrepresentation is indicated by star symbols. *n* is sample size, *p* indicates the chi-square (A) or binomial (B) tests *P*-values. *C*, The aligned sequences of artificial peptide ligands are arranged in four groups based on their relative affinities to the PSD95-PDZ1. The numbers in parentheses indicate the range of normalized phage ELISA values within a given affinity group. The ligand positions from “−4” to “−7” are boxed to indicate the area of relative concentration of positively charged residues. Arginines and lysines are highlighted green, while aspartic and glutamic acids are red. The unique i.d. numbers of artificial ligands are indicated on the left from their sequences. The digits above columns indicate the C-terminal position numbering of ligand residues. Analogous arrangements of ligands for other five PDZ domains are shown in Fig. S1A, Fig. S1B and Fig. S1C.

To delineate recognition preferences of the target PDZ domains, the last sixteen C-terminal amino acids of the peptide ligands selected in TAIS screens were analyzed using the residue-frequency-patterning (RFP) algorithm described recently [31]. The RFP procedure includes an analysis of statistical biases in relative frequencies of amino acid residues within a given set of peptide sequences followed by a search for patterns in the positioning of over-(under-)represented residues within individual peptides. The observed-to-expected ratios of individual amino acid frequencies within the whole set of artificial ligands are shown in Fig. 2A.

The relative frequencies of four residues, valine, arginine, threonine and serine are twice as high as expected. Since the probability of such frequencies arising by chance is vanishingly low (about 2.8E-15, assuming Bernoulli trials approximation), it is fair to hypothesize that V, R, T and S are the ligand residues that are preferred at the domain-ligand interaction interface and thus are likely to be important for binding to the target PDZ domains. The relative overrepresentation of V, T and S does not come as a surprise, as the known minimal recognition consensus of the PSD95 PDZ domains is X-(S/T)-X-(V/I/L)-COOH [18]. However, the essential role of the ligand's arginines in the SAP PDZ domain recognition has not been described previously. Analysis of the distribution of overrepresented residues within the last sixteen amino acids of artificial ligands (Fig. 2B) reveals that arginines are concentrated mainly at the ligand positions from “−4” to “−7”, suggesting important contributions of these positions to specificity and/or affinity of the interactions studied (by convention, residues of PDZ ligands are numbered starting with the last C-terminal residue as occupying the position “0”, penultimate residue as occupying the position “−1” and so forth, moving along the ligand sequence from C- to N-terminus).

To explore the relationships between amino acid frequencies at specific ligand positions and the strength of PDZ domain-ligand interactions we arranged peptide ligands into four groups in accord with their relative affinities: 1) best binders (normalized phage ELISA signal from 0.8 to 1.0); 2) good binders (0.6 to 0.8 ELISA signal); 3) moderate binders (0.4 to 0.6 ELISA signal) and 4) weak binders (0.2 to 0.4 ELISA signal) (see Fig. 2C). Independent of affinity ranking, the “−2” and “0” positions of ligands are almost invariably occupied by threonine and valine residues, respectively. Of note are the overwhelming preference for threonine over serine at the position “−2” and the practically complete dominance of valine over other hydrophobic residues in the role of the last C-terminal residue (Fig. 2C). The same statistical biases with respect to the “−2” and “0” positions have been observed for all six domains examined (see Fig. S1A, Fig. S1B and Fig. S1C), which is suggestive that both T and V are essential for either strong or relatively weak interactions with the PDZ domains examined, and that their substitution by other amino acids is likely to be either disruptive, or, as in the case of conservative substitutions such as T ->S or V ->I or L, significantly detrimental for binding. This conclusion is consistent with the previously reported mutational analysis of a PSD95 and SAP102 natural ligand, demonstrating that the substitution of S by T at the “−2” ligand position results in at least two-fold increase in binding affinity, and that valine as the last C-terminal residue is by far superior for binding to the first two PDZ domains of PSD95 and SAP102 in comparison to isoleucine or leucine [18]. The third overrepresented residue, serine, is rather evenly dispersed along peptide ligands with the only obvious preference for the position “−2”, as expected, where it occurs more often than in other positions, albeit infrequently relative to threonine. The fourth overrepresented residue, arginine, tends to be absent within the last four C-terminal positions of peptide ligands, in all affinity groups. Instead, arginine concentrates at the ligand positions from “−4 to −7”, with noticeable preference for the position “−4” in strong binders. In addition, visual inspection of aligned peptide ligands indicates that glutamate is clearly a preferred residue at the position “−3”, even though glutamate is not an overrepresented residue overall (Fig. 2C). The significant decrease in the relative frequencies of arginine and glutamate at the ligand positions “−4” and “−3”, correspondingly, when one compares statistics of strongest versus weakest binders, suggests that the presence of arginine and glutamate in these positions is essential for strong interactions with the target PDZ domains (see Fig. S1A, Fig. S1B and Fig. S1C).

As the described positional patterns of overrepresented residues hold for all six target domains (not shown), it is fair to conclude that the general recognition consensus of the PSD95 and SAP97 PDZ domains is X-R-E-(T/S)-X-V-COOH. Indeed, this inferred consensus represents a refinement of the well-known minimal recognition consensus of the class I PDZ domains, X-(S/T)-X-(V/I/L)-COOH, first defined for the PSD95 PDZ domains through analysis of C-terminal sequences in the PSD95 interacting partners [29], [30] and later confirmed by structural and biochemical studies [16], [18], [20], [32]. However, the prediction of natural ligands of the PSD95 PDZ domains in protein databases using this refined consensus as a query poses the following problem. Consider, as an example, the affinity-sorted sets of the PSD95-PDZ1 domain ligands shown in Fig. 2C. Notice that approximately 36% of the best binders and 68% of good binders do not feature arginine at their “-4” positions, and thus the query X-R-E-(T/S)-X-V-COOH is likely to miss a very significant fraction of natural ligands in protein databases. Relaxing the consensus to X-E-(T/S)-X-V-COOH is not very helpful either, for only about 52% of the best and good binders selected in our screens feature glutamate residues at their “−3” positions (Fig. 2C). Therefore, in an attempt to extract from protein databases as many interactors of the PSD95 PDZ domains as possible, while minimizing spurious hits, we decided to consider as putative PSD95 PDZ interactors only those proteins that present at their C-termini amino acid sequences matching either a) the consensus X-E-(T/S)-X-V-COOH, b) the last four amino acids of all the best and good binders of the PSD95 PDZ domains, or c) the last four amino acid residues of the known interacting partners of the PSD95 PDZ domains reported in web-based protein interaction databases such as MINT, PPID and IntAct. The decision to focus only on the last four C-terminal positions was driven by the prevailing assumption that only the last three to four amino acids of peptide ligands are essential for PDZ domain-mediated interactions [15], [20], [21].

### Analysis of PDZ domain recognition: natural ligands

**Figure 3:**
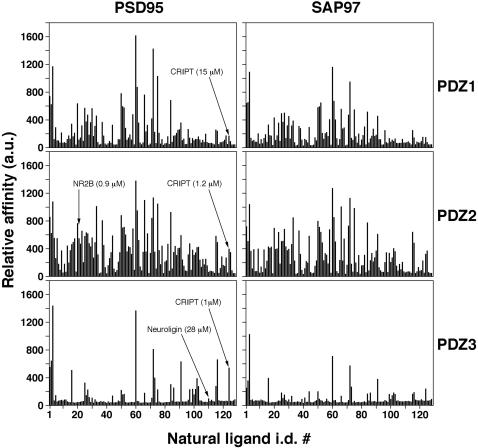
Binding of 126 proteomic fragments (natural ligands) to six PDZ domains of PSD95 and SAP97. Binding histograms were obtained by peptide ELISA performed on purified GST fusions of the indicated domains immobilized in micro-titer plate wells as described previously [31]. The X axis indicates the i.d. numbers of natural ligands (see Table S2). The Y axis indicates the peptide ELISA kinetics slope value in arbitrary units (a.u.). The 127^th^ and 128^th^ wells in each of the six sets were loaded with irrelevant peptides to indicate background signal. To illustrate internal consistency of the affinity evaluations obtained by peptide ELISA and their external consistency with the previously published affinity measurements, the reported affinities of the five PDZ domain-ligand pairs obtained by three different research groups using fluorescence polarization [17], [18], [32] are shown. The previously reported affinities provide calibration, suggesting that the individual signals that are higher than 200 a.u. roughly correspond to the interaction affinities of 15 µM *K_D_* or stronger.

A search of SWISS-PROT and TrEMBL databases with the described above queries gave 126 potential interacting partners for the PSD95 PDZ domains (see the individual C-terminal sequences of putative interactors together with their i.d. numbers in Table S2). In order to verify the predicted interactions and to evaluate relative affinities of individual natural ligands to the target domains, we synthesized 126 N-terminally biotinylated 15-mer proteomic fragments corresponding to our hits and assayed them for binding to each of the target domains *in vitro*, using the peptide ELISA assay [31] (Fig. 3).

From visual inspection of the binding histograms shown in Fig. 3, it is evident that the highest degree of promiscuity exhibited by the second PDZ domains of both proteins towards natural ligands and the more pronounced similarities in ligand preferences between homologous domains across different proteins rather than between different PDZ domains within the same protein recapitulate the patterns previously observed for artificial ligands (Fig.1). Paradoxically, however, the third PDZ domains of both proteins, PSD95 and SAP97, appear to be significantly more selective toward natural ligands than one would expect from their rather promiscuous interactions with artificial ligands (compare Fig. 1 and Fig. 3).

To pinpoint the molecular determinants in natural ligands that are responsible for strong interactions with the target PDZ domains we looked for statistical biases in the relative amino acid frequencies within the positional window “−4 to −7”, the fully degenerate positions in our queries. It is worth emphasizing that all 126 natural ligands had been selected based on their match with the last four C-terminal amino acids of artificial ligands only. In other words, if the residues upstream of the last four amino acids in natural ligands were relatively unimportant for interactions with the target PDZ domains, one would expect no significant biases in amino acid frequencies at those positions. If, on the contrary, they are both essential and specific for the target domains, then the statistical biases within this region in natural ligands should be analogous to, or at least reminiscent of, the amino acid frequency patterns observed within the same positional window in artificial ligands.

**Figure 4:**
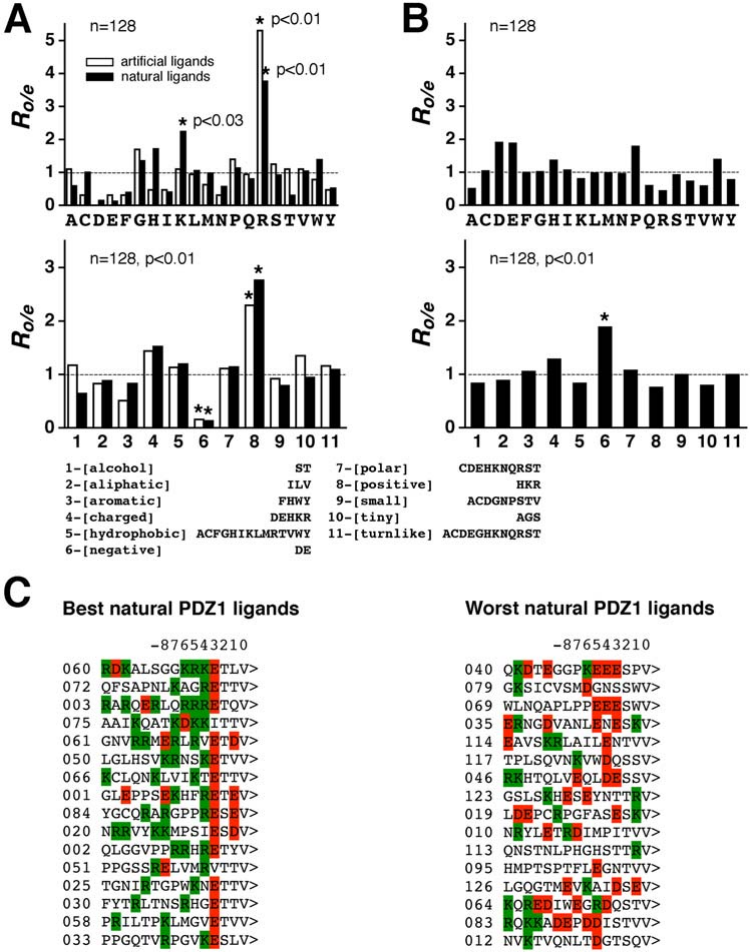
Analysis of amino acid frequency biases within the positional window “−4 to −7” in artificial and natural binders of the PSD95-PDZ1 domain. *A*, The observed-to-expected ratios of individual (top) and grouped (bottom) amino acid frequencies within the positional window “−4 to −7” in the 32 best artificial (open bars) and 32 best natural (filled bars) peptide ligands. *B*, The observed-to-expected ratios of individual (top) and grouped (bottom) amino acid frequencies within the positional window “−4 to −7” in the 32 worst natural peptide ligands. Grouping of twenty natural amino acids into eleven conserved physicochemical classes [41] is shown below histograms. Statistically significant over-(under)representation is indicated by star symbols. *n* is sample size, *p* indicates the binomial test *P*-values. *C*, Comparison of the best (on the left) and worst (on the right) natural binders of the PSD95-PDZ1 domain. The i.d. numbers of natural ligands are indicated on the left from their sequences. Arginines and lysines are highlighted green, while aspartates and glutamates are red. Notice how the relative abundance of negatively charged residues at the “−1” position in the best binders mirrors the relative abundance of positively charged residues at the “−1” position in the worst binders.

The histograms in Fig. 4 summarize and compare the amino acid compositional biases observed within the positional window “−4 to −7” of various natural and artificial ligand sets of the PSD95-PDZ1 domain. Essentially the same compositional biases were observed for natural and artificial ligands of the PSD95-PDZ2 domain and the first two SAP97 PDZ domains (not shown). Altogether, the results suggest that the positions upstream of the last four C-terminal residues in natural ligands of the first two PDZ domains do exhibit statistical biases in amino acid frequencies, and that these statistical biases do match closely the amino acid frequency patterns observed within the same positions in artificial ligands. The overrepresentation of positively charged residues, arginine and lysine, within the “−4 to −7” positional window of peptide ligands, both natural and artificial, correlates with high affinity of domain-ligand interactions, while the presence of negatively charged residues, aspartate and glutamate, within the same positional window correlates with poor or no binding at all. Noticeably, positively charged residues, especially arginine, while dispersed within the “−4 to −7” positional window in natural binders, tend to concentrate at the position “−4” in the strongest natural binders, faithfully recapitulating the arginine positional pattern observed in artificial ligands (compare Fig. 2C and Fig. 4C). Therefore, we conclude that the ligand positions “−4 to −7” are essential for selectivity and affinity of PDZ domain interactions, at least in the specific case of the first two PDZ domains of the PSD95 and SAP97 proteins. The paucity of strong natural binders for the third PDZ domains precluded analogous analysis of their natural ligands. However, the overall recognition pattern of the PSD95-PDZ3 domain is likely to be very similar to that of the first two PSD95 PDZ domains, as evidenced by the analysis of artificial ligands of the third PDZ domains discussed previously and by the comparative analysis of PSD95-PDZ2 and PSD95-PDZ3 natural ligands that follows next.

### Differential recognition by the same class PDZ domains

**Figure 5:**
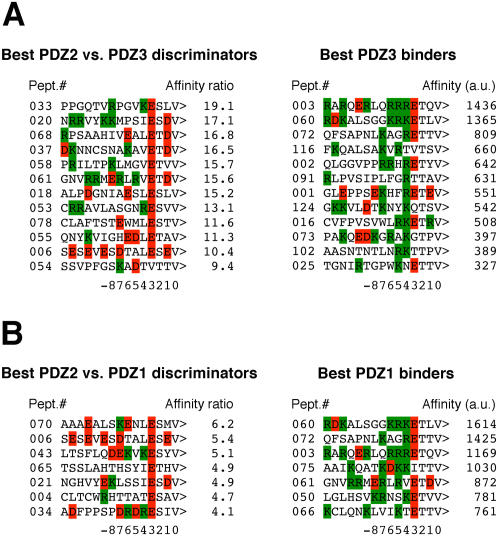
Pattern recognition differences between individual PDZ domains of PSD95. *A*, Comparison of the sequences that exhibited the highest differential ratios in their relative affinities to PDZ2 domain versus PDZ3 domain (on the left) to the sequences of the best PDZ3 binders (on the right). *B*, Comparison of the sequences that showed the highest differential ratios in their relative affinities to PDZ2 domain versus PDZ1 domain (on the left) to the sequences of best PDZ1 binders (on the right). Arginines and lysines are shown green, aspartates and glutamates are red.

In order to gain insight into the pattern recognition differences of the PDZ domains that belong to the same class but are able to differentiate between peptide ligands sharing the class-defining C-terminal consensus, we investigated a particular case of this general puzzle, namely, the differences in recognition preferences between the second and third PDZ domains of PSD95, which are classified as type I PDZ domains but are known to discriminate between various X-(S/T)-X-(V/I/L)-COOH ligands [17], [18]. In Fig. 5A we compare two sets of C-terminal sequences. The first set represents the sequences of natural ligands that bound strongly to the PSD95-PDZ2 domain but showed poor or no binding to the PSD95-PDZ3 domain. The second set shows the sequences of the PSD95-PDZ3 strongest binders. From this comparison, one can discern the following patterns: the PSD95-PDZ3 domain appears 1) to disfavor D at the “−1” ligand position; 2) to prefer T over S at the “−2” position; 3) to prefer R or K, while disfavoring I, V or L at the “−4” ligand position and 4) to favor positively charged residues in the “−4 to −7” positional window and thus, by inference, to be especially sensitive to negatively charged residues at those positions. Correspondingly, the PSD95-PDZ2 domain appears to rely on hydrophobic interactions mainly, both within the “−4 to −7” ligand positions and at the “−1” position. While a significantly larger number of different ligand sequences is needed for formal statistical validation of these tentative patterns, they are good enough to rationalize why certain PSD95 interacting partners, such as, for example, NMDA (*N*-metyl-D-aspartate) receptor subunits NR2A and NR2B with their C-terminal sequences KKMPSIESDV-COOH and EKLSSIESDV-COOH, respectively, or voltage-gated potassium channel subunits Kv1.1, Kv1.2 and Kv1.3 (C-termini VNKSKLLTDV-COOH, VNITKMLTDV-COOH and VNIKKIFTDV-COOH, correspondingly), bind to the first two PSD95 PDZ domains but fail to interact with the third PSD95 PDZ domain [18], [29], [30]. It is also likely that these patterns, tentative as they are, possess reasonable predictive power. One could hypothesize, for example, that receptor-type tyrosine protein phosphatases gamma (SWISS-PROT i.d.#P23470, C-terminus DPAESMESLV-COOH, peptide #17 in the natural ligand dataset) and zeta (SWISS-PROT i.d.# P23471, C-terminus NIAESLESLV-COOH, peptide #18 in the natural ligand dataset) would bind to the second PDZ domains of PSD95 and SAP97 but not to their third PDZ domains, as they did (Fig. 3), and so forth.

To generalize, we suggest that by increasing statistical power one is likely to detect and define robust differences in pattern recognition between any pair of individual PDZ domains. Indeed, the comparison of the natural ligands exhibiting several-fold preference, in terms of relative affinity, for the PSD95-PDZ2 domain over the PSD95-PDZ1 domain with the best binders of the latter clearly suggests that these domains are also capable of differential recognition (Fig. 5B). It appears that the accumulation of sub-optimal residues within the last 6 or 7 positions renders the first domain sensitive to the negatively charged residues at and upstream of the “−6” or “−7” positions, while the second domain, relying on hydrophobic interactions, retains significant affinity for such ligands. Speaking of methodological advantages, it should be noted that, in addition to providing high-resolution power, statistical analysis of large datasets of binders is relatively insensitive to experimental errors and variations. Perhaps even more importantly, this type of analysis is free from certain limiting assumptions implicit in more traditional approaches, thus allowing for discovery of novel unanticipated patterns. For example, the conventional “lock-and-key”-type interpretations assume independence in energetic contributions of individual ligand residues, thus implying that the inferences made from the analysis of population averages or of a few ligands are valid for all individual ligands. Whether this assumption is true or not can only be answered through systematic analysis of large populations of the “isogenic” interactions on the one-by-one basis, which have been notably lacking, mainly due to the very same assumption.

## Discussion

Summarizing the recognition patterns of the PSD95 and SAP97 PDZ domains inferred from the comparative analysis of amino acid organization of their affinity-ranked artificial and natural ligands, the PSD95 and SAP97 PDZ domains prefer positively charged residues, lysine and arginine, in the positional window “−4 to −7”, while strongly favoring lysine or arginine at the position “−4”, glutamate at the position “−3”, threonine at the position “−2” and valine at the position “0”. Even though the ligand position “−1” appears to accept various residues, it is apparently used for discrimination between individual PDZ domains – lysines and arginines are disfavored in this position by the first two PDZ domains (Fig. 4C and not shown), which is in agreement with the reported mutational analysis of the NR2B C-terminus [18], while aspartate at the “−1” position is not well tolerated by the third PDZ domain, in agreement with the mutational analysis of the CRIPT C-terminal peptide [17]. We also noticed that none of the natural binders and none of the artificial ligands feature proline at the “−4” position, suggesting a possible advantage of keeping the main chain of ligands unconstrained at this position. The relative promiscuity of the second PDZ domain can be explained by its apparent reliance on hydrophobic interactions within the positional window “−4 to −7” and, likely, at the position “−1”, which makes this domain less sensitive to lysine/arginine versus aspartate/glutamate compositional biases, thus allowing for a much greater variety of amino acids acceptable at these positions. Indeed, a number of strong binders of the second PDZ domain feature leucine, isoleucine, valine or even aspartate in the positions often occupied by lysine or arginine in the strong binders of the first and third PDZ domains (Fig. 5 and not shown).

On the whole, it appears that PDZ domain interactions are driven by the interdependent contributions of multiple ligand positions to the overall energy of interaction, which may span the last eight or more C-terminal amino acids of ligands. The unexpected plasticity and complexity of PDZ recognition are rooted in an apparently integral nature of the individual ligand residue contributions. Sub-optimal amino acids at some of the ligand positions can be compensated by optimal amino acids at other positions to preserve the strength of interaction. At the same time, even the major favorable energetic contributions of threonine and valine at the “−2” and “0” positions can be compromised by delinquent residues acting somewhere else along the chain. In the ligands featuring sub-optimal amino acids within the last four or five C-terminal ligand positions the individual contributions of upstream residues may become critical, thus allowing for highly differential recognition of such ligands by very similar PDZ domains.

The paradoxical behavior of the third PDZ domains of PSD95 and SAP97, which appear to be exquisitely selective towards natural ligands, but promiscuous toward artificial ligands, is unlikely to find its explanation in the physicochemical idiosyncrasies of the third domains only. Instead, we suggest that what appears as the exquisite selectivity of the third domain towards natural ligands may simply reflect the selective pressures imposed by evolution on functional organization of the postsynaptic density, which led to a relatively limited number of the PDZ3 ligands encoded in the genome. In this regard, a few examples from the artificial ligands dataset are most illustrative. Tryptophan is a significantly overrepresented amino acid at the “−1” position in artificial ligands, in all affinity groups, suggesting that the presence of tryptophan at the “−1” ligand position is not detrimental for interaction *per se*, with any of the target domains (Fig. 2C, Fig. S1A, Fig. S1B and Fig. S1C). If anything, it appears to be advantageous. However, only nine proteins out of the 14550 human protein entries in the SWISS-PROT database have the C-termini matching the consensus X-(S/T)-W-V-COOH. As another example, the PSD95-PDZ3 domain has selected a set of unusual peptides with the C-terminus W-Y-H-S-F-COOH (see Table S1), with which it interacts selectively and with reasonable affinity *in vitro* (Fig. 1, peptides # 17, 18, 19 and 20, and not shown). None of the human proteins appears to have such C-termini. Apparently, SAP PDZ domains are open to a much larger spectrum of interactions than that encoded in the genomic sequences.

What is then the biological meaning of adaptive plasticity in PDZ domain recognition? And what are physiologically relevant affinities of PDZ domain interactions? We speculate that both the adaptive plasticity and the wide range of interaction affinities of SAP PDZ domains are directly linked to the managerial/scaffolding role of SAP proteins in synapse organization. It is fair to suggest that both a large ligand sequence space and a wide affinity range of scaffold-mediated interactions are beneficial, if not essential, for synapse plasticity, because they define the spatio-temporal ranges within which the synapse organizational dynamics operate. The imaging studies of molecular dynamics in living cells, tissues and animals indicate that synapses [33], [34], as well as many other, and maybe all, sub-cellular structures [35], [36], are maintained as steady-state metastable molecular organizations by continuous flux of their resident components entering and leaving organizations, with individual components following individual dynamics, from very slow to very fast, as defined by specific protein associations taking place within a given sub-cellular organization. We suggest that within this image/framework of dynamic synapse, where both molecular composition and organization of the synapse at any given moment are defined to a large extent by available PDZ domain-containing scaffolds, by a particular assortment of the C-termini present at the synaptic site and by *the competition* between available C-termini for available PDZ domains, the adaptive plasticity and wide affinity range of PDZ scaffold-mediated interactions emerge as essential pre-requisites of synaptic compositional and organizational flexibility. The selectivity of PDZ domain interactions, on the other hand, ensures a certain degree of order and organizational structure required to perform synaptic functions.

We also speculate that the synaptic environmental and organizational invariants are encoded in the genome in the form of matching spectra of synapse-associated PDZ domains and their cognate ligands. In this way the genome loosely specifies the overall schematics and principles of synapse organization, while maturation, fine-tuning, and adaptation of individual synaptic structures take place as a result of their individual development and experience. In the same sense as neuronal organization of every newborn brain has been shaped by evolution to recognize certain perceptual/environmental invariants, but is not limited to recognition of those patterns only, the PDZ domains have been shaped by evolution to recognize certain C-terminal sequences present in a given proteome, but are not limited to the recognition of those sequences only. In this way, the composition, organization, and functioning of individual synapses remain open for evolution at both ontogenetic and phylogenetic levels, accommodating novel C-terminal sequences that can potentially arise from a plethora of the epigenetic and genetic molecular mechanisms known to generate molecular diversity, including posttranslational modifications, regulated proteolysis, RNA splicing, mutations, DNA rearrangements, protein splicing, and others. In short, we suggest that the adaptive plasticity of SAP PDZ domain recognition and the wide affinity range of SAP PDZ domain interactions are evolutionarily enforced by requirements of synapse plasticity and reflect the managerial role of SAP scaffolds in synaptic organizational dynamics.

It should be emphasized that the proposed conceptualization of the PDZ domain as a complex adaptive system evolved to ensure both structure and organizational flexibility of higher order macromolecular organizations not only resolves the uncertainties pertaining to PDZ domain recognition, but also suggests a fundamental molecular mechanism underlying the adaptive plasticity of sub-cellular molecular organization revealed in a number of the recent studies in which advanced imaging techniques were used to address molecular dynamics in living cells [33], [Bibr pone.0000953-Misteli1] [Bibr pone.0000953-Kurakin4] [35−37]. Specifically, we suggest that peptide recognition modules, such as PDZ, SH3, SH2, WW, EH and other domains, which show both selectivity and promiscuity in their interactions [3], [19], [24], [38], function as adaptive molecular “synapses” of cellular protein interaction networks, rather than perform as Lego Block-like elements for assembly of pre-defined and immutable structures. The combination of selectivity and plasticity may constitute inherent property of all specific protein interactions, for it has clear evolutionary advantages over mechanistic self-assembly, allowing the cell 1) to capitalize on the evolutionary memory manifested as the limited selectivity of specific protein associations, 2) to adapt its organization to a given environmental context and 3) to explore new variants of intracellular molecular organization in the course of biological evolution.

In the same sense as the overall organization of a newborn brain represents, essentially, a form of evolutionary memory, subject to both ontogenetic and phylogenetic development and maturation, the overall organization of cellular protein interaction networks encoded in the matching spectra of peptide interaction modules and their cognate ligands within a given genome may represent an evolutionary memory that is subject to ontogenetic and phylogenetic development, maturation, and adaptation (see reference [39] introducing the concept of evolutionary memory).

## Materials and methods

### Phage display libraries, peptides, proteins and antibodies

The 16-mer random peptide library was generated in-house using the T7 phage display library construction kit from Novagen. The human brain cDNA library was purchased from Novagen. The GST fusion protein expression constructs of the PSD95-PDZ2, PSD95-PDZ3, SAP97-PDZ1 and SAP97-PDZ2 domains were kindly provided by Dr. B. K. Kay (The University of Illinois at Chicago). The GST fusion constructs of the PDS95-PDZ1 and SAP97-PDZ3 domains were generated by PCR amplification of the corresponding PDZ domain coding regions from SAP cDNAs (generously provided by Dr. David S. Bredt (University of California, San Francisco)) followed by cloning into the pGEX2TK expression vector (Amersham Pharmacia). The PDZ domain borders were defined by the SMART software tools (http://smart.embl-heidelberg.de/). All the constructs were verified by sequencing. Expression and protein purification of GST fusions were performed in accord with manufacturer's instructions. The detailed protein purification protocols used in this work can be found at http://www.buckinstitute.org/TAIS. The synthetic biotinylated peptides corresponding to natural ligand sequences were purchased from JPT Peptide Technologies GmbH (Berlin, Germany). Rabbit polyclonal anti-T7 antibodies were a generously gift from Dr. F. W. Studier, Brookhaven National Laboratory. Donkey anti-rabbit antibodies conjugated to horseradish peroxidase were purchased from Amersham Biosciences.

### Target-assisted iterative screening (TAIS)

A detailed description of the TAIS method is presented in Kurakin et al. (*20*). The TAIS flowchart and protocols can be found on the Internet (http://www.buckinstitute.org/TAIS). Briefly, 30 µg of a GST-PDZ domain fusion immobilized on sepharose beads were blocked with 0.5% bovine serum albumin (BSA) in TBS-T (Tris-buffered saline, pH 7.4+0.1% Tween 20) and incubated with a phage-displayed peptide library aliquot (approx. 10^8^–10^9^ pfu). After 90 minutes of incubation at room temperature (RT) the beads were thoroughly washed with TBS-T and bound phages were eluted with 200 µl of 1% SDS for 15 min at RT. Following elution, the phages were immediately mixed with a molten 0.6% top agarose containing host cells and plated onto two pre-warmed 150 mm agar plates. When phage plaques became visible, the plates were cooled down for 30 min at 4°C and overlaid with 132 mm nitrocellulose membranes (Schleicher & Schuell) for 5 min. Following plaque lift, the membranes were blocked in 1% BSA in TBS for 1 hour at RT and incubated overnight in 25 ml of TBS-T on a rocker at 4°C with 10 µg of the target PDZ domain that had been cleaved from the GST moiety, biotinylated and complexed with streptavidin – alkaline phosphatase (STRAP) at a ratio of 4:1. After extensive washing with TBS-T, positive plaques were developed on the membranes with insoluble alkaline phosphatase (AP) substrate BCIP/NBT (Sigma). Individual positive plaques were identified on the plates and phages from these plaques were propagated separately in the appropriate host for production of individual phage lysates. The identities of phage-displayed peptides were inferred by sequencing the library-specific DNA inserts amplified by PCR from the T7 phage display vector.

### Phage ELISA

GST fusion-coated microtiter ELISA plates (COSTAR) were prepared by passive immobilization of 1 µg of the indicated GST fusion proteins (Fig. 1) per well in 200 µl of 0.1M NaHCO_3_, pH 8.0, overnight at 4°C. Following protein coating, plates were blocked by adding 150 µl of 1% BSA in TBS for 1 hour at RT. Following incubation, the wells with immobilized target proteins were washed 5 (x1ml) times with TBS-T. One hundred µl of freshly prepared individual phage lysate was added to the ELISA plate wells and incubated for 1 hour at RT. Following incubation, unbound phages were washed away with TBS-T and the amount of retained phages was determined with polyclonal T7 phage-specific antibodies followed by monoclonal anti-rabbit antibodies conjugated to horseradish peroxidase (HRP) (Amersham Pharmacia). The individual phage ELISA kinetics were followed and quantified colorimetrically using soluble HRP substrate (ABTS/H_2_O_2_). ELISA readings were taken on a SpectraMAX190 plate reader (Molecular Devices) at 405 nm. To ensure reproducibility, each of the individual phage ELISA binding histograms presented was obtained at least three times in at least three separate experiments performed by two different experimenters. The representative sets of binding histograms are shown in Fig. 1.

### Peptide ELISA

Wells of microtiter plates were coated with 1 µg of the indicated GST-PDZ domain fusions, washed with TBS-T and blocked with 1% BSA in the same way as described above for phage ELISA. Individual biotinylated peptides (30 ng) were pre-incubated with 1 µg of streptavidin-HRP conjugate (Pierce) in 300 µl of TBS-T for 30 min at RT. One hundred µl of the peptide-streptavidin-HRP conjugate were added to 100 µl of TBS-T left in each coated well after the final wash of the protein immobilization/blocking procedure. Microtiter plates were incubated for 1 hour at RT, and then washed 5 (x1mL) times with TBS-T. The amounts of peptides retained were quantified colorimetrically by adding soluble HRP substrate (ABTS/H_2_O_2_) and measuring ELISA kinetic slopes. ELISA readings were taken on a SpectraMAX190 plate reader (Molecular Devices) at 405 nm. To ensure reproducibility, all peptide ELISA experiments presented were repeated at least three times in at least three separate experiments performed by two different experimenters. The representative sets of binding histograms are shown in Fig. 3.

### Statistical analysis

Evaluation of statistical significance of amino acid frequency biases was based on the Bernoulli trials approximation, i.e. on the assumption of random and independent sampling of individual amino acids from a population with specified amino acid frequencies. In the case of artificial ligands obtained from cDNA library (about 50% of ligands) and from random peptide library (another 50%), we assumed that sampling was done from a population where individual amino acids are equally represented. We believe it is a reasonable, albeit coarse-grained, approximation both for random peptide library and for the cDNA library used, considering that about 90% of the peptides isolated from the latter represented frameshifts. It was assumed that natural ligands were sampled from a population where individual amino acid are distributed in accord with their average occurrence in the SWISS-PROT database (the v. 51.1 issue statistics). The chi-square and binomial tests *P*-values shown in the Fig. 2 and Fig. 4 were calculated and corrected for multiple testing using the GraphPad Online Calculators at http://www.graphpad.com

## Supporting Information

Figure S1A Distribution of charged residues within affinity-ranked artificial ligands of the PSD95-PDZ2 and PSD95-PDZ3 domains. The aligned sequences of artificial peptide ligands are arranged in four groups based on their relative affinities to the indicated PDZ domains. The numbers in parentheses indicate the range of normalized phage ELISA values within a given affinity group. Arginines and lysines are highlighted green, while aspartic and glutamic acids are red. Upper panel - PSD95-PDZ2 ligands; lower panel - PSD95-PDZ3 ligands.
(0.86 MB TIF)

Figure S1B Distribution of charged residues within affinity-ranked artificial ligands of the SAP97-PDZ1 and SAP97-PDZ2 domains. The aligned sequences of artificial peptide ligands are arranged in four groups based on their relative affinities to SAP PDZ domains. The numbers in parentheses indicate the range of normalized phage ELISA values within a given affinity group. Arginines and lysines are highlighted green, while aspartic and glutamic acids are red. Upper panel - SAP97-PDZ1 ligands; lower panel - SAP97-PDZ2 ligands.
(0.80 MB TIF)

Figure S1C Distribution of charged residues within affinity-ranked artificial ligands of the SAP97-PDZ3 domain. The aligned sequences of artificial peptide ligands are arranged in four groups based on their relative affinities to SAP PDZ domains. The numbers in parentheses indicate the range of normalized phage ELISA values within a given affinity group. Arginines and lysines are highlighted green, while aspartic and glutamic acids are red.
(0.41 MB TIF)

Table S1 Artificial peptide ligands isolated from phage-displayed random peptide and cDNA libraries by TAIS using various SAP PDZ domains as targets
(0.05 MB DOC)

Table S2 Putative natural peptide ligands of SAP PDZ domains
(0.06 MB DOC)
