# Supplementary figures and images for: The PDZ Domain as a Complex Adaptive System
Source: PLoS One. 2007 Sep 26;2(9):e953. doi: 10.1371/journal.pone.0000953 (PMC1978516; doi:10.1371/journal.pone.0000953)

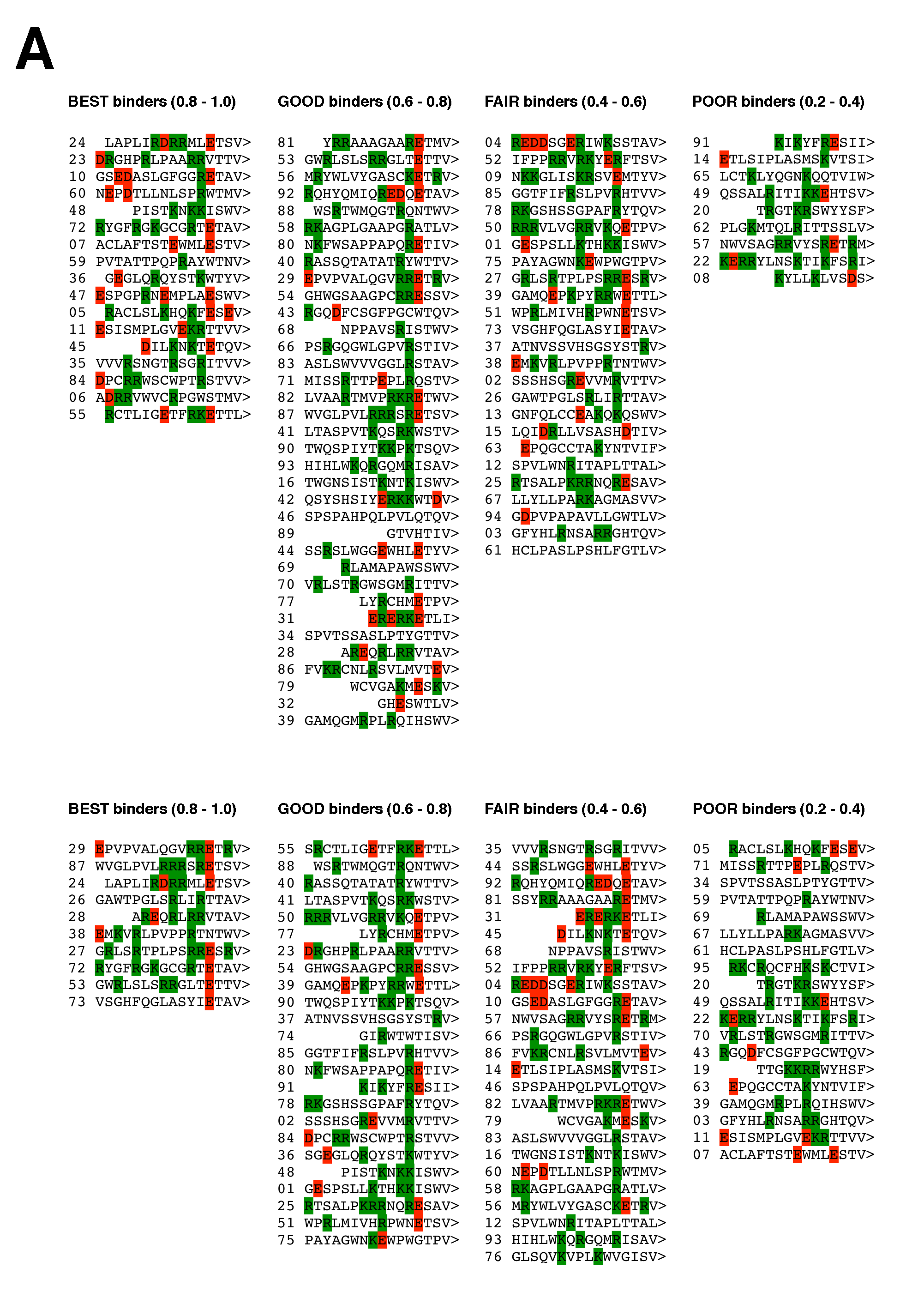

Supplement: Figure S1A — Distribution of charged residues within affinity-ranked artificial ligands of the PSD95-PDZ2 and PSD95-PDZ3 domains. The aligned sequences of artificial peptide ligands are arranged in four groups based on their relative affinities to the indicated PDZ domains. The numbers in parentheses indicate the range of normalized phage ELISA values within a given affinity group. Arginines and lysines are highlighted green, while aspartic and glutamic acids are red. Upper panel - PSD95-PDZ2 ligands; lower panel - PSD95-PDZ3 ligands. (0.86 MB TIF) [file pone.0000953.s001.tif]

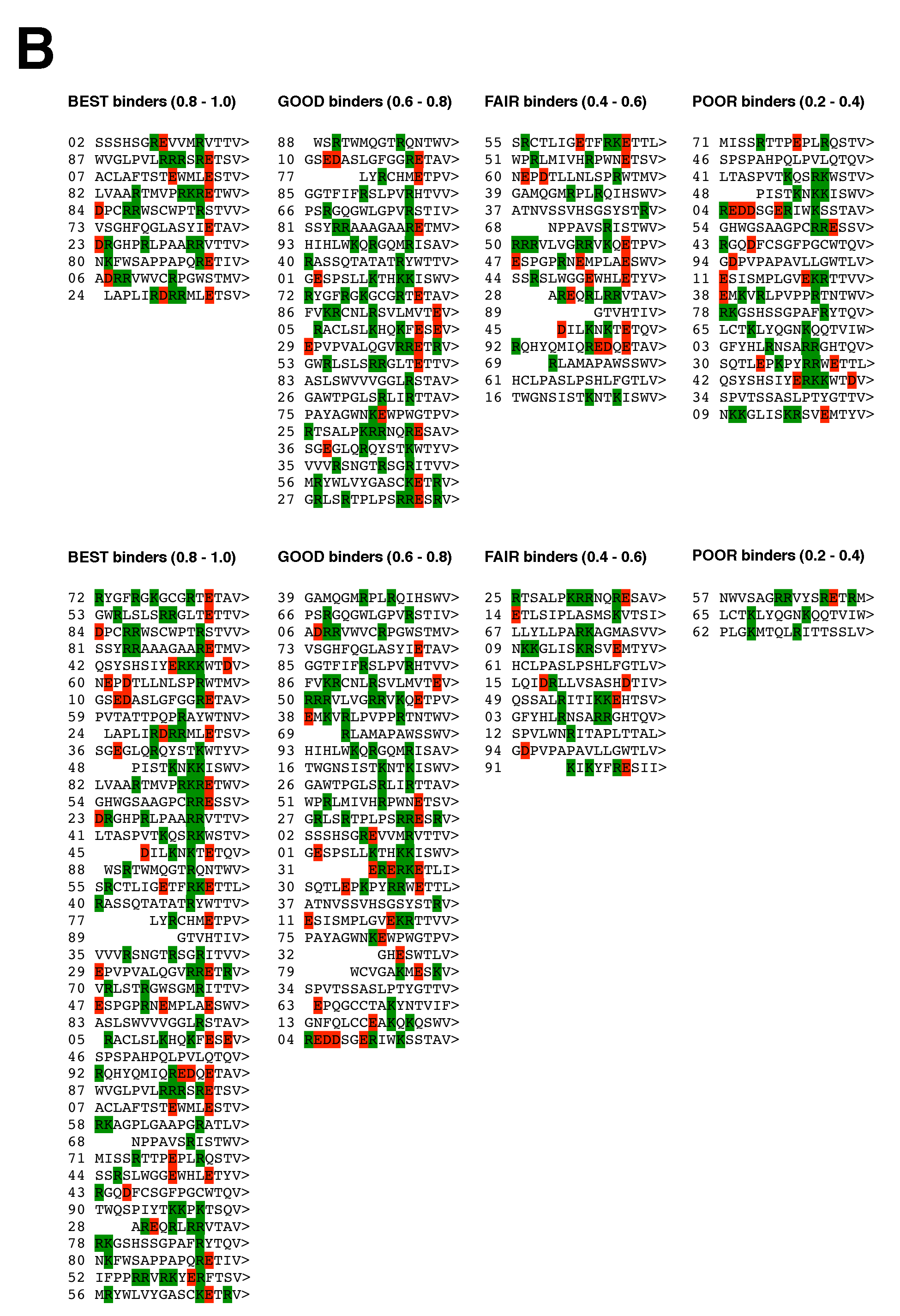

Supplement: Figure S1B — Distribution of charged residues within affinity-ranked artificial ligands of the SAP97-PDZ1 and SAP97-PDZ2 domains. The aligned sequences of artificial peptide ligands are arranged in four groups based on their relative affinities to SAP PDZ domains. The numbers in parentheses indicate the range of normalized phage ELISA values within a given affinity group. Arginines and lysines are highlighted green, while aspartic and glutamic acids are red. Upper panel - SAP97-PDZ1 ligands; lower panel - SAP97-PDZ2 ligands. (0.80 MB TIF) [file pone.0000953.s002.tif]

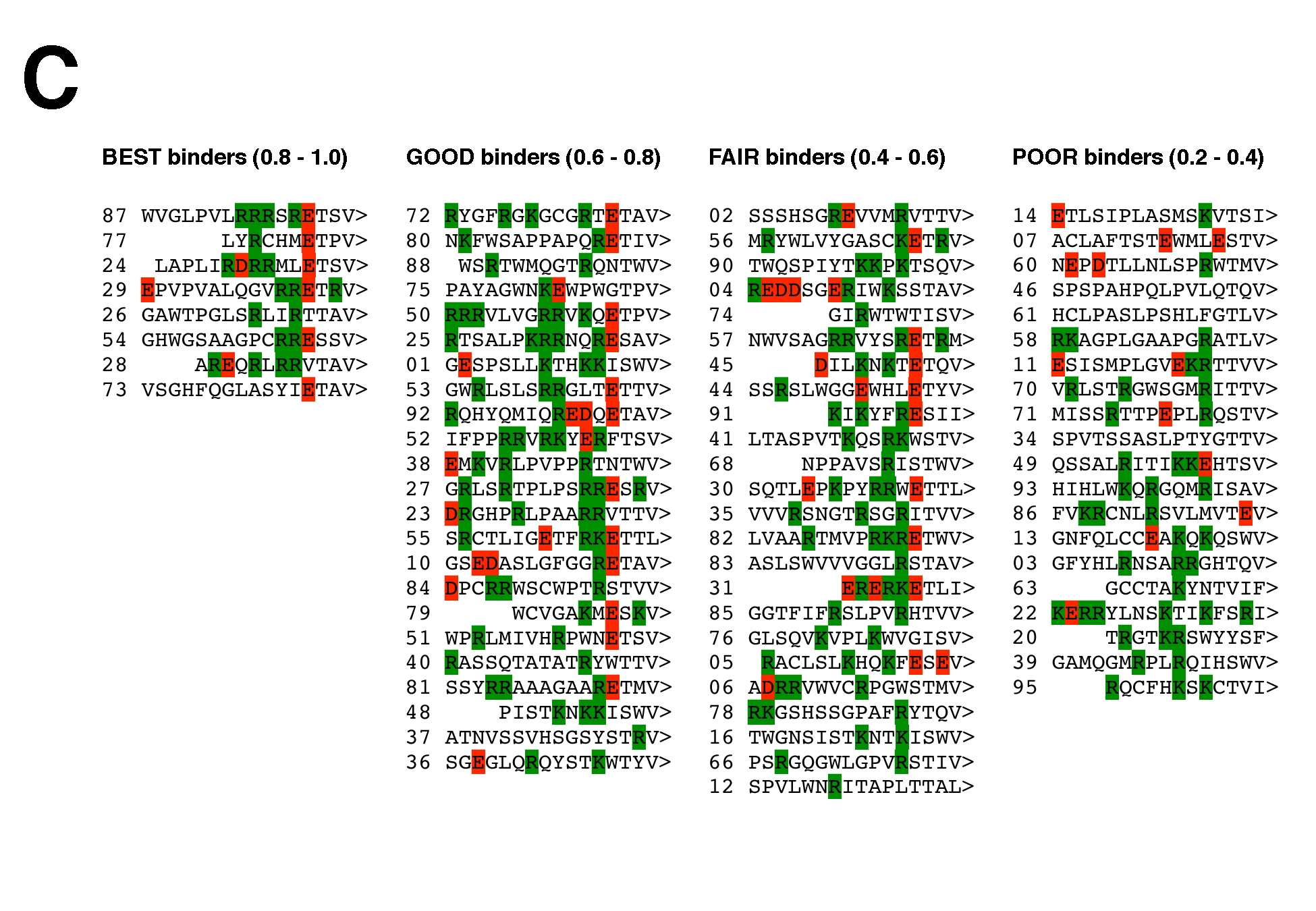

Supplement: Figure S1C — Distribution of charged residues within affinity-ranked artificial ligands of the SAP97-PDZ3 domain. The aligned sequences of artificial peptide ligands are arranged in four groups based on their relative affinities to SAP PDZ domains. The numbers in parentheses indicate the range of normalized phage ELISA values within a given affinity group. Arginines and lysines are highlighted green, while aspartic and glutamic acids are red. (0.41 MB TIF) [file pone.0000953.s003.tif]
