# Supplementary material for: The PDZ Domain as a Complex Adaptive System
Source: PLoS One. 2007 Sep 26;2(9):e953. doi: 10.1371/journal.pone.0000953 (PMC1978516; doi:10.1371/journal.pone.0000953)
Supplement: Table S1 — Artificial peptide ligands isolated from phage-displayed random peptide and cDNA libraries by TAIS using various SAP PDZ domains as targets (0.05 MB DOC) [file pone.0000953.s004.doc]

#### SUPPORTING INFORMATION

Table S1.

Artificial peptide ligands isolated from phage-displayed random peptide and cDNA libraries by TAIS using various SAP PDZ domains as targets

| i.d. # | sequence* | T7 library** |
| --- | --- | --- |
| 01 | GESPSLLKTHKKISWV> | cDNA |
| 02 | SSSHSGREVVMRVTTV> | cDNA |
| 03 | GFYHLRNSARRGHTQV> | cDNA |
| 04 | REDDSGERIWKSSTAV> | cDNA |
| 05 | RACLSLKHQKFESEV> | cDNA |
| 06 | ADRRVWVCRPGWSTMV> | cDNA |
| 07 | ACLAFTSTEWMLESTV> | cDNA |
| 08 | KYLLKLVSDS> | cDNA |
| 09 | NKKGLISKRSVEMTYV> | cDNA |
| 10 | GSEDASLGFGGRETAV> | cDNA |
| 11 | ESISMPLGVEKRTTVV> | cDNA |
| 12 | SPVLWNRITAPLTTAL> | cDNA |
| 13 | GNFQLCCEAKQKQSWV> | cDNA |
| 14 | ETLSIPLASMSKVTSI> | cDNA |
| 15 | LQIDRLLVSASHDTIV> | cDNA |
| 16 | TWGNSISTKNTKISWV> | cDNA |
| 17 | TRGTKRSWYHSF> | cDNA |
| 18 | TSGTKRSWYHSF> | cDNA |
| 19 | TTGKKRRWYHSF> | cDNA |
| 20 | TRGTKRSWYYSF> | cDNA |
| 21 | GRSPEVRSSRPVWSTW> | cDNA |
| 22 | KERRYLNSKTIKFSRI> | cDNA |
| 23 | DRGHPRLPAARRVTTV> | 16-mer random |
| 24 | LAPLIRDRRMLETSV> | 16-mer random |
| 25 | RTSALPKRRNQRESAV> | 16-mer random |
| 26 | GAWTPGLSRLIRTTAV> | 16-mer random |
| 27 | GRLSRTPLPSRRESRV> | 16-mer random |
| 28 | AREQRLRRVTAV> | 16-mer random |
| 29 | EPVPVALQGVRRETRV> | cDNA |
| 30 | SQTLEPKPYRRWETTL> | cDNA |
| 31 | ERERKETLI> | cDNA |
| 32 | GHESWTLV> | cDNA |
| 33 | ICSKNILQK> | cDNA |
| 34 | SPVTSSASLPTYGTTV> | cDNA |
| 35 | VVVRSNGTRSGRITVV> | 16-mer random |
| 36 | GEGLQRQYSTKWTYV> | 16-mer random |
| 37 | ATNVSSVHSGSYSTRV> | 16-mer random |
| 38 | EMKVRLPVPPRTNTWV> | 16-mer random |
| 39 | GAMQGMRPLRQIHSWV> | 16-mer random |
| 40 | RASSQTATATRYWTTV> | 16-mer random |
| 41 | LTASPVTKQSRKWSTV> | 16-mer random |
| 42 | QSYSHSIYERKKWTDV> | cDNA |
| 43 | RGQDFCSGFPGCWTQV> | cDNA |
| 44 | SSRSLWGGEWHLETYV> | cDNA |
| 45 | DILKNKTETQV> | cDNA |
| 46 | SPSPAHPQLPVLQTQV> | cDNA |
| 47 | ESPGPRNEMPLAESWV> | cDNA |
| 48 | PISTKNKKISWV> | cDNA |
| 49 | QSSALRITIKKEHTSV> | cDNA |
| 50 | RRRVLVGRRVKQETPV> | 16-mer random |
| 51 | WPRLMIVHRPWNETSV> | 16-mer random |
| 52 | IFPPRRVRKYERFTSV> | 16-mer random |
| 53 | GWRLSLSRRGLTETTV> | 16-mer random |
| 54 | GHWGSAAGPCRRESSV> | 16-mer random |
| 55 | RCTLIGETFRKETTL> | 16-mer random |
| 56 | MRYWLVYGASCKETRV> | 16-mer random |
| 57 | NWVSAGRRVYSRETRM> | 16-mer random |
| 58 | RKAGPLGAAPGRATLV> | cDNA |
| 59 | PVTATTPQPRAYWTNV> | cDNA |
| 60 | NEPDTLLNLSPRWTMV> | cDNA |
| 61 | HCLPASLPSHLFGTLV> | cDNA |
| 62 | PLGKMTQLRITTSSLV> | cDNA |
| 63 | EPQGCCTAKYNTVIF> | cDNA |
| 64 | AALGVPQMQMKSTTVW> | cDNA |
| 65 | LCTKLYQGNKQQTVIW> | cDNA |
| 66 | PSRGQGWLGPVRSTIV> | 16-mer random |
| 67 | LLYLLPARKAGMASVV> | 16-mer random |
| 68 | NPPAVSRISTWV> | 16-mer random |
| 69 | RLAMAPAWSSWV> | 16-mer random |
| 70 | VRLSTRGWSGMRITTV> | 16-mer random |
| 71 | MISSRTTPEPLRQSTV> | 16-mer random |
| 72 | RYGFRGKGCGRTETAV> | 16-mer random |
| 73 | VSGHFQGLASYIETAV> | 16-mer random |
| 74 | GIRWTWTISV> | 16-mer random |
| 75 | PAYAGWNKEWPWGTPV> | 16-mer random |
| 76 | GLSQVKVPLKWVGISV> | 16-mer random |
| 77 | LYRCHMETPV> | 16-mer random |
| 78 | RKGSHSSGPAFRYTQV> | 16-mer random |
| 79 | WCVGAKMESKV> | 16-mer random |
| 80 | NKFWSAPPAPQRETIV> | 16-mer random |
| 81 | YRRAAAGAARETMV> | 16-mer random |
| 82 | LVAARTMVPRKRETWV> | 16-mer random |
| 83 | ASLSWVVVGGLRSTAV> | 16-mer random |
| 84 | DPCRRWSCWPTRSTVV> | 16-mer random |
| 85 | GGTFIFRSLPVRHTVV> | 16-mer random |
| 86 | FVKRCNLRSVLMVTEV> | 16-mer random |
| 87 | WVGLPVLRRRSRETSV> | 16-mer random |
| 88 | WSRTWMQGTRQNTWV> | 16-mer random |
| 89 | GTVHTIV> | 16-mer random |
| 90 | TWQSPIYTKKPKTSQV> | cDNA |
| 91 | KIKYFRESII> | cDNA |
| 92 | RQHYQMIQREDQETAV> | cDNA |
| 93 | HIHLWKQRGQMRISAV> | cDNA |
| 94 | GDPVPAPAVLLGWTLV> | cDNA |
| 95 | RKCRQCFHKSKCTVI> | cDNA |

* “ > “ denotes carboxy-terminal group

** only the last 16 amino acid residues are shown for phage-displayed cDNA products
