# Supplementary material for: The PDZ Domain as a Complex Adaptive System
Source: PLoS One. 2007 Sep 26;2(9):e953. doi: 10.1371/journal.pone.0000953 (PMC1978516; doi:10.1371/journal.pone.0000953)
Supplement: Table S2 — Putative natural peptide ligands of SAP PDZ domains (0.06 MB DOC) [file pone.0000953.s005.doc]

#### SUPPORTING INFORMATION

**Table S2.**

**Putative natural peptide ligands of SAP PDZ domains**

| i.d. # | sequence* | SWISS-PROT i.d. |
| --- | --- | --- |
| 001 | GLEPPSEKHFRETEV> | Q9IWU6 |
| 002 | QLGGVPPRRHRETYV> | Q6RX94 |
| 003 | RARQERLQRRRETQV> | Q8V9K7 |
| 004 | CLTCWRHTTATESAV> | P36815 |
| 005 | QPLFAHIRKRGESRV> | Q8V0P1 |
| 006 | SESEVESDTALESEV> | Q69092 |
| 007 | QPLFAHLRRRGESNV> | Q9PYA6 |
| 008 | HTYTTPPYDDAETYV> | P03200 |
| 009 | ETATKVQTRANVTTV> | Q7T619 |
| 010 | NRYLETRDIMPITVV> | P13158 |
| 011 | GTLKIVCPLCTTTAV> | Q705H9 |
| 012 | NVKTVQNLTDGTSQV> | Q6XD88 |
| 013 | LERVIFPSVKIATLV> | P89079 |
| 014 | QPPVHADPPAGGTTV> | Q9WS61 |
| 015 | TGWKHERKLSSESQV> | O75096 |
| 016 | CVFPVSVWLRKETRV> | Q9NS75 |
| 017 | IADESDPAESMESLV> | P23470 |
| 018 | ALPDGNIAESLESLV> | P23471 |
| 019 | LDEPCRPGFASESKV> | P08588 |
| 020 | NRRVYKKMPSIESDV> | Q12879 |
| 021 | NGHVYEKLSSIESDV> | Q13224 |
| 022 | GPCTWRRISSLESEV> | Q14957 |
| 023 | TRRGSAHFSSLESEV> | O15399 |
| 024 | TGPLNLSDPSVSTVV> | Q5VSF5 |
| 025 | TGNIRTGPWKNETTV> | Q5T234 |
| 026 | GGCMKTGLWKSETTV> | Q96PE1 |
| 027 | TGNVRTGLWKHETTV> | Q8IWK6 |
| 028 | PTRVSFHSIKQSTAV> | P34998 |
| 029 | FYTRLTNSKQGETTV> | Q9UP38 |
| 030 | FYTRLTNSRHGETTV> | Q14332 |
| 031 | GNGWVKPGKGSETVV> | Q9ULV1 |
| 032 | FYHRLSHSSKGETAV> | Q9ULV1 |
| 033 | PPGQTVRPGVKESLV> | P35499 |
| 034 | ADFPPSPDRDRESIV> | Q14524 |
| 035 | ERNGDVANLENESKV> | P48051 |
| 036 | EQNGCLPPPESESKV> | Q92806 |
| 037 | DKNNCSNAKAVETDV> | P22459 |
| 038 | PPLWAPPGKHLVTEV> | Q96RP8 |
| 039 | LNAFEEVEEFPETSV> | Q8NET8 |
| 040 | QKDTEGGPKEEESPV> | P53985 |
| 041 | PEKNGEVVHTPETSV> | O15427 |
| 042 | LNHCTIQISELETNV> | O00341 |
| 043 | LTSFLQDEKVKESYV> | O95477 |
| 044 | LPQSDSSLQSLETSV> | P23634 |
| 045 | QRQPLPDSNPEESSV> | Q9C0C4 |
| 046 | RKHTQLVEQLDESSV> | Q9NTN9 |
| 047 | RGVGGSAERLEESVV> | Q6PJG9 |
| 048 | SLHIPFVETQHQTQV> | Q8TDW7 |
| 049 | LAAGLLKKVAEETPV> | Q9P0N8 |
| 050 | LGLHSVKRNSKETVV> | Q86UD3 |
| 051 | PPGSSRELVMRVTTV> | Q9P2E8 |
| 052 | SSHSGREVVMRVTTV> | Q86VN5 |
| 053 | CRRAVLASGNRESVV> | Q9NVW2 |
| 054 | SSVPFGSKADTVTTV> | Q5T2K1 |
| 055 | QNYKVIGHEDLETAV> | O75912 |
| 056 | QHYQMIQREDQETAV> | Q13574 |
| 057 | LGVLIYEMLVGETSV> | Q5TBX8 |
| 058 | PRILTPKLMGVETVV> | Q9Y6W6 |
| 059 | TDSSCGNSRHGESNV> | Q9NR81 |
| 060 | RDKALSGGKRKETLV> | Q5SQI7 |
| 061 | GNVRRMERLRVETDV> | Q86TF0 |
| 062 | NVERMGRLLGLETNV> | Q6AZ96 |
| 063 | LLSTDNPDALPETLV> | O43182 |
| 064 | KQREDIWEGRDQSTV> | Q7Z5L2 |
| 065 | TSSLAHTHSYIETHV> | Q8WXI2 |
| 066 | KCLQNKLVIKTETTV> | Q6PDA6 |
| 067 | LLKGRTACCHSETVV> | P01135 |
| 068 | RPSAAHIVEALETDV> | Q96KB5 |
| 069 | WLNQAPLPPEEESWV> | Q8NHY3 |
| 070 | AAAEALSKENLESMV> | Q9UI32 |
| 071 | LLFEMLCALPKVTTV> | Q9BSE5 |
| 072 | QFSAPNLKAGRETTV> | Q4VXC3 |
| 073 | PAKQEDKGRAKGTPV> | P82930 |
| 074 | VIGGDHKLLFWVTEV> | Q8NFH4 |
| 075 | AAIKQATKDKKITTV> | Q96A65 |
| 076 | TSPIKPKRPFVESNV> | Q9NXY9 |
| 077 | VTHDKNTCIIYESHV> | O75121 |
| 078 | CLAFTSTEWMLESTV> | Q8TBS9 |
| 079 | GKSICVSMDGNSSWV> | Q8NGB0 |
| 080 | DGKRVYNPLLSVTTV> | Q76G19 |
| 081 | DGTRVYNSFLSVTTV> | Q9UPQ7 |
| 082 | RGTFGSSEWVMESTV> | Q9ULH4 |
| 083 | RQKKADEPDDISTVV> | Q96PB8 |
| 084 | YGCQRARGPPRESEV> | Q8TAY7 |
| 085 | SKLEEKHIQVPESIV> | O95727 |
| 086 | KSHKFVLRLQSETSV> | Q8TAA9 |
| 087 | RQKDNCNTVTVETVV> | Q2TBC9 |
| 088 | SVGTSQELLEYETTV> | Q5GH72 |
| 089 | ITDKKCSRQQVESRV> | Q6JV82 |
| 090 | SANPKAKPSRISTVV> | Q5JU85 |
| 091 | RLPVSIPLFGRTTAV> | Q8TB68 |
| 092 | TRGSQDLGNRSATLV> | Q9NPB5 |
| 093 | ADRDHLQPAVFGTTV> | Q2NL98 |
| 094 | TAQINIGHNPGSSSV> | Q69016 |
| 095 | HMPTSPTFLEGNTVV> | O95136 |
| 096 | TVLPPPPYRHRNTVV> | Q15303 |
| 097 | RSLSSFKLAKKSSSV> | Q8NG42 |
| 098 | TFKDTANLVKEDSEV> | P98164 |
| 099 | DSLHSNTANRRTTPV> | Q9Y698 |
| 100 | ESLHNNPANRRTTPV> | O60359 |
| 101 | EGFHVSMLNRRTTPV> | Q9UBN1 |
| 102 | AASNTNTLNRKTTPV> | Q8WXS5 |
| 103 | DSLHSNTANRRTTPV> | Q2M1M1 |
| 104 | ANQNCVNKSKLLTDV> | Q09470 |
| 105 | ANTNYVNITKMLTDV> | P16389 |
| 106 | NPNSCVNIKKIFTDV> | P22001 |
| 107 | SAPDSATDSQLQSSV> | Q96BD0 |
| 108 | PGGGRHSRSSLNTVV> | P98153 |
| 109 | RPRVRLGSEIRDSVV> | Q9NPR2 |
| 110 | PHPHPHPHSHSTTRV> | Q8N2Q7 |
| 111 | SHNNTLPHPHSTTRV> | Q8NFZ4 |
| 112 | FNSTGLPHSHSTTRV> | Q9NZ94 |
| 113 | QNSTNLPHGHSTTRV> | Q8N0W4 |
| 114 | EAVSKRLAILENTVV> | P21941 |
| 115 | QSPKRHSGSYLVTSV> | P25054 |
| 116 | FKQALSAKVRTVTSV> | Q9BPW5 |
| 117 | TPLSQVNKVWDQSSV> | O14578 |
| 118 | PRWHLGPGDPFSTYV> | O60229 |
| 119 | VYVGGKQVVPFSSSV> | Q9Y2T3 |
| 120 | EKAEDGSSSKEGTSV> | Q9P2E9 |
| 121 | ATTDTLESTTVGTSV> | Q15046 |
| 122 | NVWQVNASRTRITFV> | P33993 |
| 123 | GSLSKHESEYNTTRV> | Q8TDF5 |
| 124 | GKKVLDTKNYKQTSV> | Q9P021 |
| 125 | GSSPSHSATSVHTSV> | Q13613 |
| 126 | LGQGTMEVKAIDSEV> | Q9P1G9 |

* “ > “ denotes carboxy-terminal group
